# Tobacco Smoking, Alcohol Drinking, Diabetes, Low Body Mass Index and the Risk of Self-Reported Symptoms of Active Tuberculosis: Individual Participant Data (IPD) Meta-Analyses of 72,684 Individuals in 14 High Tuberculosis Burden Countries

**DOI:** 10.1371/journal.pone.0096433

**Published:** 2014-05-02

**Authors:** Jayadeep Patra, Prabhat Jha, Jürgen Rehm, Wilson Suraweera

**Affiliations:** 1 Centre for Global Health Research, St Michael's Hospital, Toronto, Canada; 2 Dalla Lana School of Public Health, University of Toronto, Toronto, Canada; 3 Centre for Addiction and Mental Health, Toronto, Canada; McGill University, Canada

## Abstract

**Background:**

The effects of multiple exposures on active tuberculosis (TB) are largely undetermined. We sought to establish a dose-response relationship for smoking, drinking, and body mass index (BMI) and to investigate the independent and joint effects of these and diabetes on the risk of self-reported symptoms of active TB disease.

**Methods and Findings:**

We analyzed 14 national studies in 14 high TB-burden countries using self-reports of blood in cough/phlegm and cough lasting > = 3 weeks in the last year as the measures of symptoms of active TB. The random effect estimates of the relative risks (RR) between active TB and smoking, drinking, diabetes, and BMI<18.5 kg/m^2^ were reported for each gender. Floating absolute risks were used to examine dyads of exposure. Adjusted for age and education, the risks of active TB were significantly associated with diabetes and BMI<18.5 kg/m^2^ in both sexes, with ever drinking in men and with ever smoking in women. Stronger dose-response relationships were seen in women than in men for smoking amount, smoking duration and drinking amount but BMI<18.5 kg/m^2^ showed a stronger dose-response relationship in men. In men, the risks from joint exposures were statistically significant for diabetics with BMI<18.5 kg/m^2^ (RR = 6.4), diabetics who smoked (RR = 3.8), and diabetics who drank alcohol (RR = 3.2). The risks from joint risk factors were generally larger in women than in men, with statistically significant risks for diabetics with BMI<18.5 kg/m^2^ (RR = 10.0), diabetics who smoked (RR = 5.4) and women with BMI<18.5 kg/m^2^ who smoked (RR = 5.0). These risk factors account for 61% of male and 34% of female estimated TB incidents in these 14 countries.

**Conclusions:**

Tobacco, alcohol, diabetes, and low BMI are significant individual risk factors but in combination are associated with triple or quadruple the risk of development of recent active TB. These risk factors might help to explain the wide variation in TB across countries.

## Introduction

The World Health Organization (WHO) estimated that 8·6 million people developed new tuberculosis (TB) infection and close to 1 million died of it in 2012 [1]. Over 95% of TB deaths occur in low- and middle-income countries (LMIC), and 22 high burden countries account for 82% of annual new cases globally [1].

Various studies and meta-analyses of published studies [2]–[6] have shown a strong association between tobacco smoking and TB infection, active TB disease or TB mortality. A smaller number of meta-analyses have noted the association between active TB disease and alcohol drinking [7], [8], diabetes mellitus [9], and low Body Mass Index (BMI) [10]. These risk factors may help predict the wide variation in TB prevalence and mortality across countries [11]. However, these meta-analyses have not explored the effects of multiple exposures on active TB disease. For example, many adult males in LMICs smoke tobacco and drink alcohol. These interactions can be measured using individual patient data (IPD), but not easily from summary measures from published studies. Nationally representative studies enable reliable projections for an entire country of the proportion of TB attributed to these various risk factors. We conducted IPD meta-analyses of standardized national surveys in 14 high-TB burden countries to establish a dose-response relationship for smoking, drinking, and BMI and to investigate the independent and joint effects of tobacco, alcohol use, diabetes and BMI<18.5 kg/m^2^ on the risk of self-reported symptoms of active TB disease.

## Methods

### Ethics

An ethics statement was not required for this work.

### Data

WHO implemented the World Health Surveys (WHS) between 2002 and 2004 in 70 countries [12] including 14 of the 22 high-TB burden countries (India, China, South Africa, Pakistan, Bangladesh, Philippines, Ethiopia, Democratic Republic of Congo, Viet Nam, Myanmar, Russia, Kenya, Brazil, Zimbabwe; ranked by no of TB cases), which collectively cover about 70% of estimated global annual new TB cases. WHS household samples were drawn from nationally representative sampling frames [13] except in China, India, and in Russia, where the surveys were done in specific regions. One adult aged 18+ years was randomly selected from each eligible household. We focused on ages 18–54 years, during which about two-thirds of all global TB cases or TB mortality occur [1].

The main outcome was the presence of self-reported symptoms of active TB disease, as defined by a positive answer to two questions ‘Over the last 12 months, have you coughed blood or had blood in your phlegm?’ and ‘Over the last 12 months, have you coughed that lasted for 3 weeks or longer?’. Four exposures were studied. Current smoking was defined as daily or occasional smoking. Most smoked products were of cigarettes, except in South Asia, where bidis were more common. As cessation is uncommon in most of these countries [14], current smoking approximated ever smoking in most countries. Among current smokers, daily consumption was classified as 1–10, 11–20, 21+ times per day, and the duration as 1–10, 11–20, and 21+ years of daily smoking. Drinking was defined as ever versus never drinkers. Drinkers were asked how many standard drinks (12 grams of pure alcohol was used as one standard drink) were consumed per day in the last week from which we defined the dose of drinking as 1–2, 3+ standard drinks per day. Diabetes was defined as those individuals who reported yes to either of two questions: ‘Have you ever been diagnosed with diabetes (high blood sugar)?’ and ’Have you ever had any treatment or medications or attended a program for diabetes?’ BMI was calculated from self-reported body weight and height. Low BMI was defined as <18.5 kg/m^2^ with dose-response analysis examining BMI scores of <16·0 kg/m^2^, 16·0–16·99 kg/m^2^, 17–18·49 kg/m^2^.The WHS did not include mycobacterial culture or sputum smear microscopy confirmation or any biological testing to confirm diabetes or smoking status.

### Statistical analysis

We used logistic regression to relate the odds ratios, as an approximation of the relative risk (RR) and the corresponding 99% confidence intervals (CI) for self-reported symptoms of active TB disease. Analyses were stratified by gender and adjusted for age (in linear years), completed years of education (none, 1–5, 6–10, or 11+ years), and where relevant, ever/never smoking or alcohol use, presence of diabetes and BMI<18.5 kg/m^2^. We used floating absolute risks to compare more than two categories of the various dyads of exposures. Floating absolute risks do not change the RRs, but the confidence intervals are assigned an appropriate standard error (the coefficient of variation of the RR) to the logRR estimates including reference category with RR = 1 to capture each level's random variation [15].

For dose-response estimates, where available, we used the midpoint of the following categories: duration of smoking, number of cigarettes or bidis or pipes, number of standard drinks and BMI score (cut off scores of 15 to 60 kg/m^2^). Sensitivity analyses compared pooled summary estimates for subgroups stratified on quality-associated study characteristics Including Directly Observed Therapy – Short course (DOTS) coverage over 75% and step by step adjustments for age, education, and where applicable smoking, alcohol use, diabetes and BMI<18.5 kg/m^2^.

To estimate gender specific alcohol-, tobacco-, diabetes- and BMI<18·5 kg/m**^2^**-attributable new TB incident cases in 14 HBCs, the Population Attributable Fractions (PAF) were applied to WHO's estimation of new TB cases in each country for 2012 [1]. PAF was calculated as P(RR−1)/(1+P(RR−1)). R refers to pooled RR based on 14 countries derived from the current meta-analysis and P refers to the prevalence in each country, drawn from independent surveys of tobacco [16], [17], alcohol use [16], [18], diabetes [19], and BMI <18·5 kg/m**^2^** [16], [20]. Where independent surveys were not available, we used age and gender specific WHS survey prevalence [21] among those not reporting a history of active TB symptoms.

The DerSimonian and Laird random-effects method was used to combine the natural logarithm of the adjusted odds ratios across countries. Where a survey provided dose-response analysis data (e.g., smoking, drinking and BMI), the risk estimates for each category were pooled using the inverse variance weighted method to derive a single estimate for each range of dose. We conducted a meta-regression using linear as well as first-order and second-order fractional polynomials to estimate the best fitting curve. Statistical heterogeneity between studies was assessed using I^2^ statistic. All analyses were done using STATA12·1.

## Results

A total of 72,684 respondents aged 18+ years were included from 14 countries (Table 1) of which 58,933 respondents were age 18–54 years (Men: 27,102; Women: 31,831). More women (34%) than men (21%) had less than primary education. Among men, 50% smoked tobacco, 37% drank alcohol, 15% had BMI <18·5 kg/m^2^ and 2% reported diabetes; among women, 9% smoked tobacco, 14% drank alcohol, 20% had <18·5 kg/m^2^ and 2% reported diabetes. About 1·4% of men and 1·2% of women self-reported symptoms of active TB disease in the last year. Inexplicably, women surveyed in India and Bangladesh reported much higher prevalences of smoking than reported in recent nationally-representative surveys [17]. The gender-specific prevalences of drinking alcohol, diabetes and BMI <18·5 kg/m^2^ were generally consistent with those reported from global reviews of the country-specific data [16], [18], [19], [22], [23].

**Table 1:** Background characteristics of respondents in World Health Surveys among high TB- burden countries, 2002-4 (18–54 years)*.

Country†	Population in 2010 (10^3^)	# people surveyed	Currently married, %		Less than primary, %		Rural residence, %		Smoking, %		Drinking, %		Low BMI (<18.5), %		Diabetes, %		TB^£^, %	
			M	W	M	W	M	W	M	W	M	W	M	W	M	W	M	W
India	1 224 614	8205	74·0	82·5	30·6	55·6	90·3	89·1	49·6	13·5	19·3	1·5	30·5	35·6	2·7	1·7	2.2	1.2
China	1 341 335	2511	81·9	86·3	6·8	13·8	69·8	68·6	59·5	1·4	44·7	9·2	4·9	10·5	0·9	1·4	0.2	0.3
South Africa	50 133	2031	40·1	33·1	16·0	18·4	39·2	47·4	40·4	14·5	42.0	17·3	25·3	26·9	5·1	8·7	1.3	1.5
Pakistan	173 593	5437	68·4	81·3	43·7	72·8	68·4	63·9	31·9	5·1	0·4	0.0	13·3	16·1	1·4	2·4	1.2	1.3
Bangladesh	148 692	4697	76·0	83·6	50·5	61·5	69·8	77·5	63·2	25·2	14·4	0·4	17·8	17·4	3·2	3·3	1.7	1.3
Philippines	93 261	8509	64·9	71·0	16·3	11·9	39·4	37·8	60.0	10·8	75·6	29·6	10·9	17·6	2·8	3.0	1.3	1.6
Ethiopia	82 950	4160	68·9	68·6	48·8	68·5	85·4	83·3	8·9	0·5	38·6	33·2	11.0	15·7	0·3	0·1	2·5	2·3
DR Congo	65 966	2196	26·8	30·2	17·3	23·9	5·1	6·0	15·6	2·1	41·4	34·2	6·7	8·7	1·5	1·4	0.8	0.6
Viet Nam	87 848	2957	78·0	78·3	13·3	19·6	80·0	76·2	57·3	2.0	64·4	4·4	16.0	27.0	0·3	0·4	0.4	0.1
Myanmar	47 963	4710	58·1	61·7	34·6	44·1	72·1	69·9	49·9	13·1	34·4	2·1	13·7	16·3	0·5	0·3	0.3	0.2
Russia	142 958	2525	54·2	54·1	0·5	0·3	13·9	11·6	59·9	19·1	88·9	77·7	1.0	4.0	1.0	1·1	0.3	0.5
Kenya	40 513	3675	60·9	66·9	28·0	41·8	81·9	82·2	27·8	1·6	39·6	11.0	14·3	10·1	0·8	1.0	1.8	1.0
Brazil	194 946	3901	45·3	43·4	21·5	21·0	17·0	16·3	28·4	20·2	88·4	71·8	2·9	6·9	3·2	5·3	2.0	1.9
Zimbabwe	12 571	3419	58·9	65·1	12·4	25·9	62·5	65·6	24·4	1·7	42·1	6·3	4·8	4·5	0·8	1.0	2.4	2.4
**14 HBCs** ¶	**3 707 343**	**58 933**	**72·9**	**77·4**	**21·2**	**33·9**	**70·9**	**68·3**	**50·6**	**9.0**	**36·8**	**14·2**	**15·2**	**19·6**	**1·9**	**2·2**	**1.4**	**1.2**

* Samples were nationally representative except in China, India, and the Russian Federation, where the survey was carried out in geographically limited regions;

† Countries are ranked by TB incidence.

£ Presence of symptoms of active TB disease was based on self-reports of blood in cough or phlegm and self-reports of coughs lasting more than 3 weeks in last 12 months.

¶ Proportions are gender specific weighted average using respective population; M: men; W: women.

### Risks and Quality assessment

Ever smoking, ever drinking, diabetes history, or BMI<18·5 kg/m^2^ were each independently associated with the risks of self-reported symptoms of active TB (Table 2). Adjusted for age and education, the risks of self-reported symptoms of active TB were significantly associated in men for diabetes (RR = 2.9), followed by BMI<18·5 kg/m^2^ (RR = 2·3), ever drinking (RR = 1·3), and non-significantly associated with ever smoking (RR = 1·2). Among women, the corresponding risks were significantly associated for diabetes (RR = 2·7), then BMI<18·5 kg/m^2^ (RR = 2·3), ever smoking (RR = 2·0) and ever drinking (RR = 1·5). These risks showed little downward attenuation after additional adjustment for the other risk factors. A history of diabetes in men, which showed increased risk with adjustment. The risks for the four exposures were comparable in settings where DOTS coverage surpassed 75% and was below 75%. Risks were slightly greater for those reporting a history of diabetes than those reporting treatment for diabetes in men, but were similar in women. The heterogeneity of the risks across countries (as defined by the I^2^ statistic) was below 20%, with the exception of ever drinking among women.

**Table 2:** Quality assessment and stratification: tobacco smoking, alcohol use, diabetes, low Body Mass Index (BMI) and risk of self-reported symptoms of active Tuberculosis (TB) disease in HBCs, 18–54 years.

Risk factors	Adjustments	Men					Women				
		Exposed TB+/Total	Non-exposed TB+/Total	Pooled Est.	99% CI	I^2^ (surveys)	Exposed TB+/Total	Non-exposed TB+/Total	PooledEst.	99% CI	I^2^ (surveys)
Ever smoking	A	178/11,690	209/15,058	1.09	0.71–1.89	0.0% (14)	59/2434	336/29,079	2.14	1.71–3.48	0.3% (9)
	A+E			1.16	0.64–2.13	0.0% (14)			2.02	1.52–3.11	0.0% (9)
	A+E+Alc			1.07	0.74–1.87	0.0% (14)			1.93	1.35–2.70	0.0% (9)
	A+E+Alc+DM+LBMI			1.14	0.72–1.91	0.0% (14)			2.11	1.43–3.40	0.0% (8)
Ever drinking	A	174/11,302	211/15,146	1.28	1.03–1.81	0.0% (13)	97/6094	296/25,169	1.31	0.91–1.96	25.1% (10)
	A+E			1.26	1.01–1.73	0.0% (13)			1.50	1.10–2.24	17.1% (10)
	A+E+Alc			1.19	0.95–1.62	0.0% (13)			1.31	0.88–1.89	10.3% (10)
	A+E+T+DM+LBMI			1.11	0.81–1.67	0.0% (12)			1.28	0.78–1.67	2.2% (10)
Diabetes	A	18/513	370/26,483	2.59	1.51–5.80	0.0% (8)	19/679	376/31,055	2.41	1.46–4.12	0.0% (8)
	A+E			2.87	1.44–5.69	0.0% (8)			2.68	1.35–5.32	0.0% (8)
	A+E+Alc+T			3.11	1.51–6.37	18.2% (8)			2.31	1.31–4.32	0.0% (8)
	A+E+Alc+T+LBMI			3.78	1.82–7.56	17.1% (8)			2.54	1.56–5.39	0.0% (7)
Low BMI	A	85/3106	213/18,637	2.30	1.64–3.23	0.0% (13)	76/4138	197/19,567	2.44	1.61–3.31	0.0% (12)
	A+E			2.27	1.61–3.20	0.0% 13)			2.32	1.55–2.91	0.0% (12)
	A+E+Alc+T			2.29	1.63–3.23	0.0% 13)			2.18	1.60–3.26	0.0% (12)
	A+E+Alc+T+DM			2.35	1.67–3.34	0.0% 13)			2.28	1.56–3.45	0.0% (12)

TB^+^ referred to active TB disease; A: Age; E: Education, Alc: ever alcohol use, T: current tobacco smoking; DM: Self-reported diabetes; LBMI: Low BMI.

### Dose-response relationships

**Figure 1:**
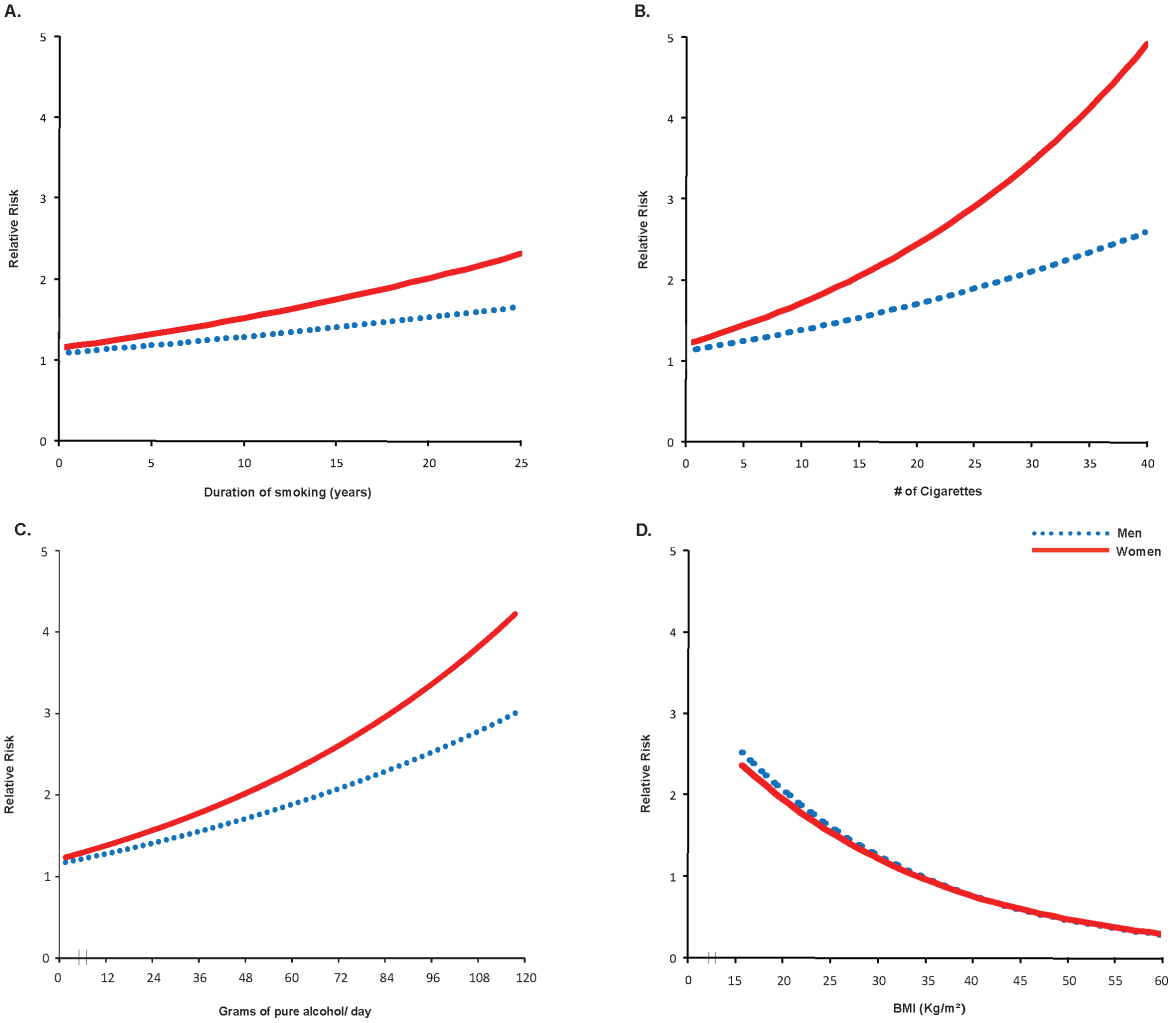
Dose-response relationships of (A) duration of smoking, (B) quantity of smoking, (C) quantity of alcohol consumption, and (D) BMI with self-reported symptoms of active TB disease by gender.

After adjustment for confounders, smoking amount, smoking duration and drinking amount showed a stronger dose response relationship in women than in men; however BMI<18·5 kg/m^2^ showed a stronger dose-response relationship in men (Table 3). Substantial risks of self-reported symptoms of active TB were seen in men and especially in women for 21 or more years of smoking (Men RR = 1·6; Women RR = 2·0), 21 or more cigarettes per day (Men RR = 2·2, Women RR = 3·0), for 3 or more standard drinks (Men RR = 1·3, Women RR = 3.2) and for BMI<16·0 kg/m^2^ (Men RR = 3.5, Women RR = 2.7). Use of linear units to measure smoking and alcohol quantity showed similar findings to the categorical results with a weaker association for the duration of smoking. Smoking amount, smoking duration, and drinking amount showed a steeper dose-response relationships in women than in men (Figure 1). By contrast, BMI<17·0 kg/m^2^ showed similar yet stronger (RR>2·1) relationships in both men and women. The heterogeneity of the risks across countries was below 20%, with the exception of number of cigarettes per day and number of standard drinks in both men and women.

**Table 3:** Dose-response relationship between current smoking, current alcohol drinking, Body Mass Index (BMI) and risk of self-reported symptoms of active TB disease in HBCs, 18-54 years.

Category*	Exposure level	Men					Women					
		TB^+^/Total	TB Prev.	Pooled Est.¶	99% CI	I^2^	TB^+^/Total	TB Prev.	Pooled Est.¶	99% CI	I^2^	
**Duration of current daily smoking†**	Never smoker	209/15,058	1.4%	1·00	0·81–1·23	*0·0%*	316/28,298	1.1%	1.00	0·83–1·20	*0·0%*	
	*1*–*10 years*	71/4817	1.5%	*1.06*	*0.74*–*1.52*	*0·0%*	37/1315	2.8%	*2.78*	*1.65*–*4.70*	*0·0%*	
	*11*–*20 years*	45/2804	1.6%	*1.16*	*0.76*–*1.78*	*3.1%*	9/648	1.4%	*1.38*	*0.53*–*3.57*	*0·0%*	
	*> = 21 years*	34/1574	2.2%	*1.57*	*1.00*–*2.54*	*0·0%*	13/471	2.8%	*2.04*	*0.82*–*5.08*	*0·0%*	
**Test for trend of odds:**			**χ2 = 7.1, p<0·008**					**χ2 = 10.5, p<0·002**				
**Daily no. of cigarettes/pipes†**	Never smoker	209/15,058	1.4%	1.00	0·87–1·21	*0·0%*	316/28,298	1.1%	1.00	0·81–1·23	*0·0%*	
	*1*–*10*	84/5673	1.4%	*1.09*	*0.62*–*1.93*	*0·0%*	45/1971	2.3%	*1.71*	*1.12*–*2.61*	*17·0%*	
	*11*–*20*	38/2685	1.4%	*1.09*	*0.57*–*2.09*	*32.4%*	11/383	2·9%	*2.52*	*1.13*–*5.63*	*71.5%*	
	*> = 21*	28/837	3.3%	*2.19*	*1.08*–*4.44*	*0·0%*	3/80	3.8%	*2.96*	*0.64*–*13.62*	*0·0%*	
**Test for trend of odds:**			**χ2 = 10.1, p<0·002**					**χ2 = 13.5, p<0·0001**				
**Daily no. of standard drinks‡**	Abstainer	211/15,126	1.4%	1.00	0·82–1·23	*0·0%*	296/25,155	1.2%	1.00	0·86–1·16	*0·0%*	
	*1*–*2 std. drinks/day*	150/9896	1.5%	*1.25*	*0.94*–*1.66*	*34.1%*	91/5906	1.5%	*1.58*	*1.08*–*2.31*	*0·0%*	
	*3+ std. drinks/day*	24/1459	1.6%	*1.31*	*0.74*–*2.30*	*0·0%*	7/213	3.3%	*3.16*	*0.84*–*11.89*	*23·0%*	
**Test for trend of odds:**			**χ2 = 3·9, p<0·01**					**χ2 = 9.3, p<0·003**				
**BMI (kg/m^2^)**	18.5 and over	204/18,469	1.1%	1.00	0·79–1·19	*0·0%*	192/19,357	1.0%	1.00	0·79–1·17	*0·0%*	
	17.0–18.49	37/1855	2.0%	1.65	1.01–2.67	*0·0%*	35/2371	1.5%	1.51	0.93–2.45	*0·0%*	
	16.0–16.99	22/566	3.9%	3.40	1.95–5.94	*0·0%*	14/774	1.8%	1.78	0.86–3.68	*0·0%*	
	<16.0	26/674	3.9%	3.47	1.91–6.30	*0·0%*	26/961	2.7%	2.66	1.53–4.63	*0·0%*	
**Test for trend of odds:**			**χ2 = 70.8, p<0·001**					**χ2 = 29·6, p<0·001**				

* adjusted for age and level of education; self-reported alcohol use†, current smoking‡, diabetes, and BMI<18.5 kg/m^2^ were additionally adjusted in the respective model;

¶ 99% CIs shown in the reference categories indicate floating absolute risks (FARs).

### Interactions of smoking, drinking, diabetes and low BMI

**Figure 2:**
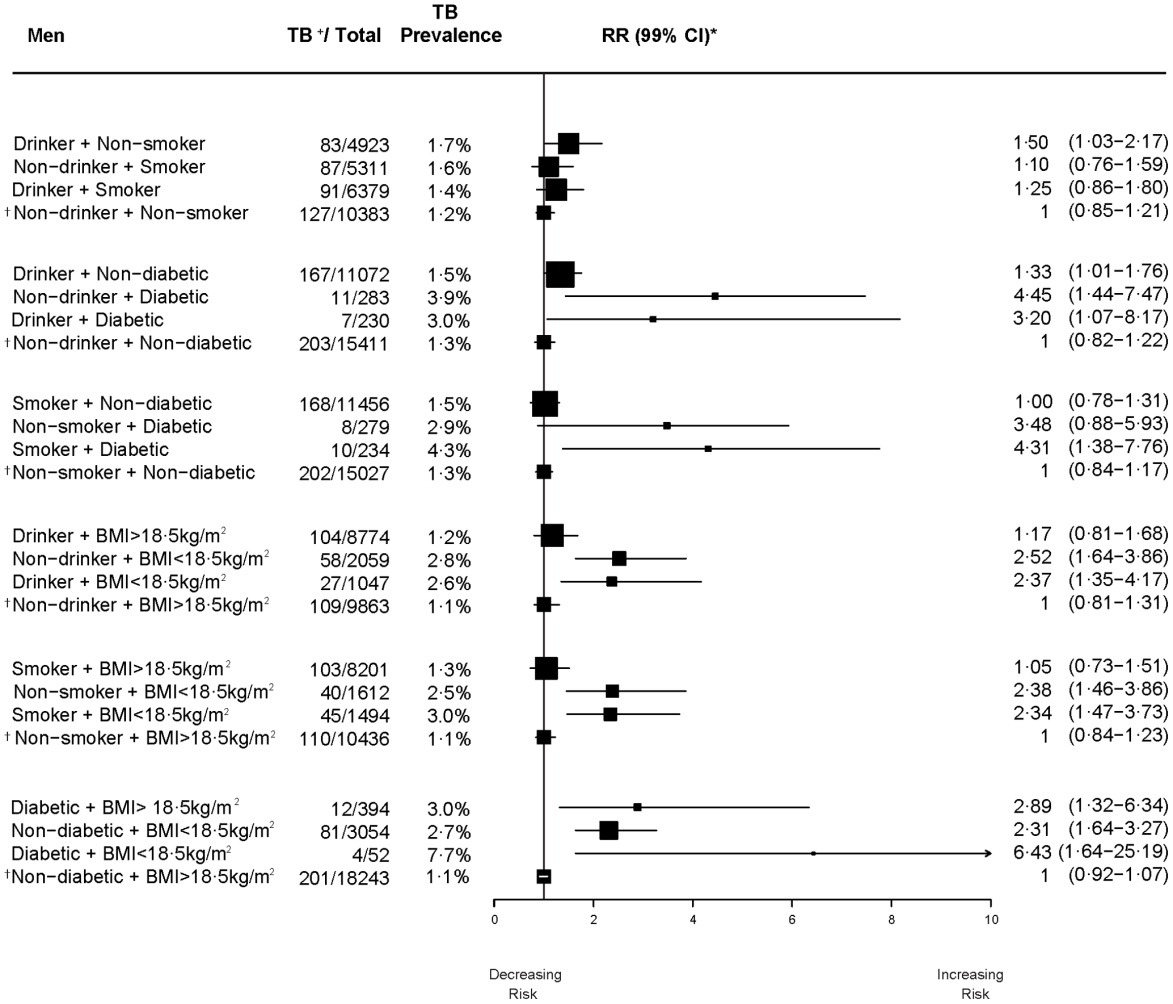
Independent and joint effects of risk factors on self-reported symptoms of active TB disease, 18–54 years in men.

**Figure 3:**
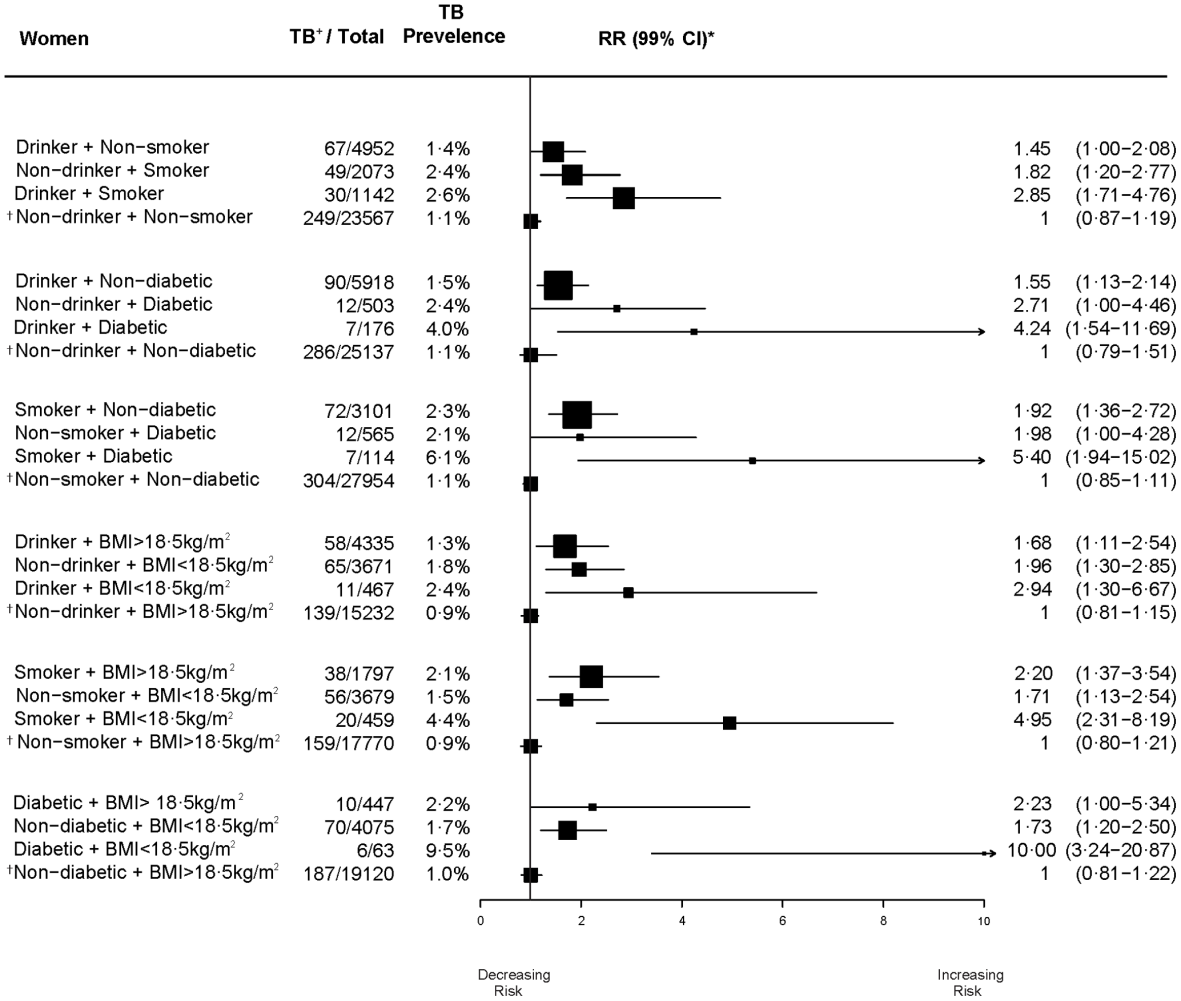
Independent and joint effects of risk factors on self-reported symptoms of active TB disease, 18–54 years in women.

Drinking and smoking were closely correlated in men; about 38% of the control men (6,288/16,352) without any history of self-reported symptoms of active TB reported both drinking alcohol and smoking tobacco. Among women, reported drinking and smoking was less common (14%; 1,112/8,021; Figure 2). By contrast the other dyads of diabetes, drinking, smoking, and BMI<18·5 kg/m**^2^** were less common in men and women. The floating absolute risks of the six dyads of exposure, comparing independent and interactive relative risks (smoking, drinking, diabetes, and BMI<18·5 kg/m^2^), were adjusted for age and education (see Figures 2 & 3). In men, the risks of self-reported symptoms of active TB for individual risk factors were statistically significant for those reporting a history of diabetes who did not drink alcohol (RR = 4.5), diabetics who did not smoke tobacco (RR = 3.5), diabetics with BMI> = 18·5 kg/m**^2^** (RR = 2.9; but with large heterogeneity across the countries), and those with BMI<18·5 kg/m**^2^** who did not drink alcohol (RR = 2.5) (Figure 2). In men, the risks from joint exposures were statistically significant diabetics with BMI<18·5 kg/m**^2^** (RR = 6.4), for diabetics who smoked (RR = 4.3, with large heterogeneity across countries), and diabetics who drank alcohol (RR = 3·2). Much lower individual and joint risks were seen for smoking or drinking in men. The numbers of cases in men and women were small for the dyads related to diabetes and BMI<18·5 kg/m**^2^**, and the confidence intervals were wide. Similarly, the small number of cases precluded the ability to detect statistically significant interactions of joint exposures.

In women, the risks of self-reported symptoms of active TB for individual risk factors were generally lower than for men, with the exception of smoking among women who did not drink alcohol (Figure 3). In women, the most notable individual risks for self-reported symptoms of active TB were for diabetics who did not drink alcohol (RR = 2.7), diabetics with BMI> = 18·5 kg/m**^2^** (RR = 2·2), smokers with BMI>18·5 kg/m**^2^** (RR = 2·2), diabetics who did not smoke (RR = 2·0), or women with BMI<18·5 kg/m**^2^** who did not drink alcohol (RR = 2.0). The risks from joint risk factors were generally larger in women than in men, with the greatest risks for diabetics with BMI<18·5 kg/m**^2^** (RR = 10.0), diabetics who smoked (RR = 5.4) and those with BMI<18·5 kg/m**^2^** who smoked (RR = 5.0). There was little heterogeneity in these individual and joint risks across countries for women.

### Risk factor contribution to TB incidence in 14 high-burden TB countries

These relative risks, adjusted for age and education might be causally important for prevalent active TB (although the reverse causality of TB causing low BMI cannot be excluded). If these measured risks are causally important, then it is possible to estimate indirectly their contribution to WHO's estimate of incidence cases occurring in these 14 high-burden TB countries. Incident TB cases are estimated to be 3·9 and 2 million in men and women, respectively in these countries in 2012 (Table 4). Among men, BMI<18·5 kg/m^2^ accounted for 17% of incident TB cases (653,000/year), followed by daily smoking (16%; 629,000/year), drinking of 40+ grams/day of alcohol (15%; 569,000/year), and diabetes (13%; 508,000/year), and. Among women, BMI<18·5 kg/m^2^ accounted for 15% of incident TB cases (304,000/year), followed by diabetes (11%; 221,000/year), daily smoking (5%; 102,000/year) and drinking of 20+ grams/day (2%; 65,000/year). In these 14 high TB burden countries, all 4 risk factors combined contributed more than one-third of global TB burden (See Table S1 for country-wise estimates).

**Table 4:** Smoking, drinking, diabetes, BMI<18.5 kg/m^2^ attributable new TB incident cases among men and women (18+ years) in 14 High-Burden Countries (HBCs), 2012.

Sex	New cases in HBC	Smoking*, %		Drinking†, %		Diabetes ‡, %		BMI<18.5 kg/m^2^ ¶, %	
**Men**	3,893,910	629,304	16	569,304	15	508,066	13	652,534	17
**Women**	2,045,090	102,076	5	65,111	3	221,011	11	303,614	15
**M+W**	**5,939,000**	**731,380**	**12**	**634,415**	**11**	**729,077**	**12**	**956,148**	**16**

* Sources of prevalence for daily smoking were taken from Global Adult Tobacco Survey (GATS) [17], Demographic and Health Survey (DHS) [16], World Health Survey (WHS) [21]; RR men = 1·71 (99% CI:1·20–2·70) and RR women = 2·44 (99% CI:1·54–4·17) were taken from our continuous dose-response meta-regression model.

† Prevalences for daily alcohol drinking (men: 40+ grams/day; women: 20+ grams/day) were taken from WHS [21] and where data on large nationally representative surveys were available [16], [18]; RR men = 1·84 (99% CI:1·32–2·83) and RR women = 1·61 (99% CI:1·11–2·49) were taken from our continuous dose-response meta-regression model.

‡ Prevalence on diabetes were taken from International Diabetes Federation (2011) [19]; RR men = 2.87 (99%CI:1·44–5.69) and RR women = 2·68 (99% CI: 1·35–5.32) were taken from our D-L meta analysis.

¶ Prevalence on low BMI (<18·5 kg/m^2^) were taken from DHS [16]; RR men = 2·12 (99% CI: 1·41–3.31) and RR women = 2·00 (99% CI: 1·43–3·24) were taken our continuous dose-response meta-regression model.

## Discussion

Our pooled analyses of individual participant data from standardized surveys in 14 LMIC find that smoking, heavy alcohol use, diabetes, and BMI<18·5 kg/m**^2^** are individual risk factors but combined together can triple or quadruple the risk of development of recent active TB disease. A clear dose-response relationship of increased TB risks was noted with increasing amounts of tobacco and alcohol consumption, increasing smoking duration and with decreasing BMI. Unlike previous meta-analyses, we were able to explore the joint and independent effects of various exposures. Notwithstanding the wide confidence intervals, the greatest elevated risks of self-reported symptoms of active TB for both genders, particularly for women were seen consistently with diabetes or BMI<18·5 kg/m^2^. Even within sub-groups of those who smoked or drank alcohol diabetes and BMI<18·5 kg/m^2^ significantly raised risks of self-reported symptoms of active TB.

### Causal mechanisms

WHO estimates that about one-third of the people in the world are infected with *Mycobacterium tuberculosis* [24] with 90% being asymptomatic latent TB infections [25]. Smoking, drinking, diabetes and low BMI might each plausibly enable progression from latent to active TB disease. Possible mechanisms for smoking include the impaired clearance of secretions on the tracheobronchial mucosal surface [26], reduced phagocytic function of pulmonary alveolar macrophages [27], decreased production of tumor necrosis factor in pulmonary macrophages [28], and increased iron overload in pulmonary macrophages [29]. Chronic alcohol use has been shown to reduce macrophage response to immune system modifiers raising the risk of acquiring disease [30], [31]. Experimental studies find that hyperglycemia may affect the host immune response to TB [32], [33]. BMI<18·5 kg/m^2^ is a possible marker for under-nutrition [34], [35], which can reduce host protective immune response either by interfering in the interaction between monocyte-macrophages and T-lymphocytes and their cytokines [36], or by secondary immunodeficiency that increases the host susceptibility to infection [37].

These risk factors account for 61% of male and 34% of female estimated incident TB cases in these 14 countries. The consistency of the relative risks suggests that worldwide, these risk factors might play an important role in explaining the wide country-specific variation in TB. Notably, the inter-relation of low BMI and tobacco use might well help to explain the high levels of TB infection and mortality in India [38]. A joint effect of tobacco use and BMI has already been reported for all-cause mortality [39] in India. The cross-sectional nature of the analysis cannot exclude if TB infection leads to low BMI, even though earlier longitudinal studies have shown that low BMI increased subsequent risk of developing active TB [35], [40]. Micronutrient intake has also been shown to decrease the risk of reversion of sputum culture to positivity after initial conversion in the first month in both HIV-infected and non-infected patients [41]. A randomized trial found that nutritional counseling with provision of supplements in TB patients produced a short term increase in body weight, lean mass, and physical function [42].

About 1·4% of men and 1·3% of women without self-reported symptoms of active TB reported having both diabetes and BMI<18·5 kg/m^2^, consistent with some reports in LMICs [43], [44]. By contrast, in most high income countries diabetes is associated with high BMI (and prolonged adiposity can be a cause of diabetes [45]). Adiposity and diabetes prevalences are rising overall in many LMICs [46], [47], and their possible interaction with smoking or heavy alcohol drinking on active TB disease is of concern.

### Limitations and Strengths

The study results are broadly representative of many countries where TB infection remains common and are based on standardized methods. Results showed consistency across countries with little attenuation with adjustment for possible confounding variables, and clear dose-response relationships. There are, however, a few potential limitations to this study. First, confounding by variables might be associated with each of the exposures. Stratification by quality of study indicators, and adjustment for education found only slight reductions in the risks, except diabetes in men, which increased with adjustment. Second, microbiological confirmation of TB was not possible as the WHS did not include mycobacterial culture or sputum smear microscopy. An affirmative response to questions on bloody cough or phlegm might indicate those with pneumonia or lung cancer, although lung cancer would tend to occur at older ages than our specified range of 18–54 years. The ongoing Million Death Study [48] in India found that the vast proportion of those reporting a history of blood in phlegm had a final diagnosis of tuberculosis, with far less due to pneumonia and even fewer due to lung cancer. Although not every participant with these TB symptoms will have microbiologically confirmed TB case, yet studies show self-reported TB has a sensitivity between 65 to 70% and specificity between 55 to 75% in comparison to sputum testing [49], [50]. Third, the assessment of risk factors were also based on self-reports only. For example, diabetes was not measured on test of fasting-plasma glucose. However, earlier studies suggest sensitivity and specificity of these with self-reported diabetes are between 65 to 85% and 95 to 99%, respectively, compared with biochemical markers, with overall Kappa coefficients above 0·80 [51], [52]. The assessment of tobacco smoking, drinking alcohol or height/weight measurements relied on self-reported behavior, which may not have been accurate due to recall or reporting bias, especially among those who consider smoking or drinking to be socially prohibitive or stigmatizing, such as women in some cultural settings. However, this would tend to make our estimates more conservative. The prevalence of smoking among those self-reporting bloody cough/phlegm and chronic cough was lower than that reported in other studies of tuberculosis disease [4]–[6]. This might reflect under-reporting of smoking by those with recent symptoms of active active TB. Indeed higher prevalences of smoking are reported among TB deaths by living household respondents [53] from whom under-reporting of smoking is less likely than from self-reports.

### Implications

TB patients generally lack access to smoking cessation services, and smoking is associated with worse outcomes for TB treatment [54], [55]. Most smoking-related immunologic abnormalities appear to be reversible within weeks of cessation. Thus, cessation might yield substantial positive effects on TB treatment outcomes, relapse and future lung disease [56], [57]. Our findings suggest that TB control programs might consider targeting patients with diabetes for interventions such as active case finding and the treatment of latent TB and, conversely, those efforts to diagnose, detect, and treat diabetes may have a beneficial impact on TB control [58]. The TB control programs in China and India recently introduced bidirectional screening of TB and diabetes [59], [60].

These findings suggest the need to adopt stricter health policies, most notably higher taxation to reduce smoking and heavy drinking in various populations where TB infection, disease and mortality remain common [61], [62]. Prospective studies are required to document if these risk factors account for the wide variation in TB disease across various countries.

## Supporting Information

Table S1 **Smoking, drinking, diabetes and BMI<18.5 kg/m^2^ attributable new TB incident cases among men and women (18+ years) in 14 High Burden Countries (HBCs), 2012.**
(PDF)

Checklist S1 **PRISMA Checklist used in this study.**
(DOC)
