# Supplementary material for: Tobacco Smoking, Alcohol Drinking, Diabetes, Low Body Mass Index and the Risk of Self-Reported Symptoms of Active Tuberculosis: Individual Participant Data (IPD) Meta-Analyses of 72,684 Individuals in 14 High Tuberculosis Burden Countries
Source: PLoS One. 2014 May 2;9(5):e96433. doi: 10.1371/journal.pone.0096433 (PMC4008623; doi:10.1371/journal.pone.0096433)
Supplement: Table S1 — Smoking, drinking, diabetes and BMI<18.5 kg/m2 attributable new TB incident cases among men and women (18+ years) in 14 High Burden Countries (HBCs), 2012. (PDF) [file pone.0096433.s001.pdf]

Table S1. Smoking, drinking, diabetes and BMI<18.5 kg/m^2 attributable new TB incident cases among men and women (18+ years) in 14 High Burden Countries (HBCs), 2012

HBCs†	MEN								WOMEN							
	Smoking*		Drinking†		Diabetes‡		BMI<18.5 kg/m^2 ¶		Smoking*		Drinking†		Diabetes‡		BMI<18.5 kg/m^2 ¶	
	TB cases	PAF	TB cases	PAF	TB cases	PAF	TB cases	PAF	TB cases	PAF	TB cases	PAF	TB cases	PAF	TB cases	PAF
India	190,453	13%	126,868	8%	215,636	14%	346,530	23%	28,470	4%	5,077	1%	79,004	11%	128,848	19%
China	170,722	24%	160,811	22%	112,313	16%	62,644	9%	7,132	3%	5,072	2%	35,538	13%	19,428	7%
South Africa	34,586	12%	66,836	23%	31,714	11%	25,861	9%	21,543	9%	17,328	7%	23,159	10%	18,726	8%
Pakistan	36,554	17%	-	-	25,034	12%	57,647	27%	13,064	7%	-	-	18,911	10%	44,638	23%
Bangladesh	52,963	23%	2,288	1%	31,590	14%	48,628	21%	2,967	2%	-	0%	18,325	15%	27,830	23%
Phillipines	39,262	21%	52,501	29%	24,452	13%	22,222	12%	7,766	10%	3,828	5%	9,279	12%	8,376	11%
Ethiopia	4,649	4%	24,095	19%	7,317	6%	26,578	21%	-	0%	10,391	10%	5,944	6%	21,573	21%
DR Congo	6,113	5%	20,791	18%	5,125	4%	7,996	7%	1,807	2%	10,201	11%	3,929	4%	7,640	8%
Viet Nam	21,418	22%	32,673	34%	5,000	5%	22,142	23%	606	2%	671	2%	1,534	5%	6,862	21%
Myanmar	23,419	18%	10,154	8%	14,460	11%	17,431	13%	5,703	8%	171	0%	7,040	10%	9,666	14%
Russia	26,288	28%	34,648	37%	15,714	17%	1,051	1%	7,191	20%	7,436	21%	6,083	17%	1,351	4%
Kenya	9,142	12%	12,390	17%	5,139	7%	8,501	12%	-	0%	1,280	3%	2,852	6%	4,718	10%
Brazil	7,518	12%	17,182	27%	9,563	15%	2,660	4%	4,054	14%	3,101	11%	4,105	14%	1,127	4%
Zimbabwe	6,219	15%	8,067	19%	5,009	12%	2,642	6%	1,773	4%	555	2%	5,307	15%	2,833	8%
**14 HBCs**	629,304	16%	569,304	15%	508,066	13%	652,534	17%	102,076	5%	65,111	3%	221,011	11%	303,614	15%
**Proportion to 14 TB HBCs**	16%	(...)	15%	(...)	13%	(...)	17%		5%	(...)	3%	(...)	11%	(...)	15%	(...)
**Proportion to 22 TB HBCs**	14%	(...)	12%	(...)	11%	(...)	14%		4%	(...)	3%	(...)	9%	(...)	13%	(...)
**Proportion to global TB burden**	11%	(...)	10%	(...)	9%	(...)	12%		3%	(...)	2%	(...)	7%	(...)	10%	(...)

*Sources of prevalence for daily smoking were taken from Global Adult Tobacco Survey (GATS) [17], Demographic and Health Survey (DHS)[16], World Health Survey (WHS)[21]; RR men=1·71 (99% CI:1·20–2·70) and RR women=2·44 (99% CI:1·54–4·17) were taken from our continuous dose-response meta-regression model. † Prevalences for daily alcohol drinking (men: 40+ grams/day; women: 20+ grams/day) were taken from WHS[21] and where data on large nationally representative surveys were available [16,18]; RR men=1·84 (99% CI:1·32–2·83) and RR women=1·61 (99% CI:1·11–2·49) were taken from our continuous dose-response meta-regression model. ‡ Prevalence on diabetes were taken from International Diabetes Federation (2011) [19]; RR men=2.87 (99%CI:1·44–5.69) and RR women=2·68 (99% CI: 1·35–5.32) were taken from our D-L meta analysis. ¶ Prevalence on low BMI (<18·5 kg/m^2) were taken from DHS[16]; RR men=2·12 (99% CI: 1·41–3.31) and RR women=2·00 (99% CI: 1·43–3·24) were taken our continuous dose-response meta-regression model.
